# Estimating attendance for breast cancer screening in ethnic groups in London

**DOI:** 10.1186/1471-2458-10-157

**Published:** 2010-03-25

**Authors:** Christine Renshaw, Ruth H Jack, Steve Dixon, Henrik Møller, Elizabeth A Davies

**Affiliations:** 1 King's College London, Thames Cancer Registry, 42 Weston Street, London, SE1 3QD, UK; 2 London Cancer Screening Quality Assurance Reference Centre, 4th Floor 50 Eastbourne Terrace, London, W2 6LG, UK

## Abstract

**Background:**

Breast screening uptake in London is below the Government's target of 70% and we investigate whether ethnicity affects this. Information on the ethnicity for the individual women invited is unavailable, so we use an area-based method similar to that routinely used to derive a geographical measure for socioeconomic deprivation.

**Methods:**

We extracted 742,786 observations on attendance for routine appointments between 2004 and 2007 collected by the London Quality Assurance Reference Centre. Each woman was assigned to a lower super output (LSOA) based on her postcode of residence. The proportions of the ethnic groups within each LSOA are known, so that the likelihood of a woman belonging to White, Black and Asian groups can be assigned. We investigated screening attendance by age group, socioeconomic deprivation using the Index of Deprivation 2004 income quintile, invitation type and breast screening service. Using logistic regression analysis we calculated odds ratios for attendance based on ethnic composition of the population, adjusting for age, socioeconomic status, the invitation type and screening service.

**Results:**

The unadjusted attendance odds ratios were high for the White population (OR: 3.34 95% CI [3.26-3.42]) and low for the Black population (0.13 [0.12-0.13]) and the Asian population (0.55 [0.53-0.56]). Multivariate adjustment reduced the differences, but the Black population remained below unity (0.47 [0.44-0.50]); while the White (1.30 [1.26-1.35]) and Asian populations (1.10 [1.05-1.15]) were higher. There was little difference in the attendance between age groups. Attendance was highest for the most affluent group and fell sharply with increasing deprivation. For invitation type, the routine recall was higher than the first call. There were wide variations in the attendance for different ethnic groups between the individual screening services.

**Conclusions:**

Overall breast screening attendance is low in communities with large Black populations, suggesting the need to improve participation of Black women. Variations in attendance for the Asian population require further investigation at an individual screening service level.

## Background

The primary aim of the NHS breast screening programme is to reduce deaths from breast cancer by early detection. To be effective the screening programme requires a large proportion of the population to respond and the government's minimum target for uptake is 70 percent[1]. A three year rolling programme invites women aged 50-70 for screening. The upper age limit for screening was extended in 2004 to include women aged 65 to 70 and by 2012 the age range will include those aged 47 to 73 years. Women of all ages, at high familial risk or with other genetic conditions predisposing to cancer are also to be invited for screening from 2009[2].

London has one of the lowest attendance for breast screening and in 2005-06, 62 percent of women attended their appointment, compared with the national average of 75 percent[3]. Uptake of breast screening is particularly low in the inner London areas. The accuracy of population registers and movement of the population is considered one of the main reasons for the low attendance[4]. Participation in a breast screening programme may also be influenced by factors relating to age, socioeconomic group, awareness of the programme or ethnicity. One of the reasons suggested for the low attendance in London is that the population is ethnically diverse, with 6% of the screened age group consisting of Black ethnic groups and 7% from the Asian ethnic groups estimated from the Census 2001. These compare to figures of 1.3% and 2.4% respectively, for England and Wales as a whole. A series of local analyses have suggested varying attendance among women from different ethnic groups in different areas of London[5]. A survey using a structured questionnaire in South East London investigating attitudes of women towards breast cancer found that whether they considered screening personally relevant was a predictor of attendance[6]. Black and Asian women were less likely to believe that they were personally at risk of breast cancer. A qualitative study in Hackney which included a sample of women representing the ethnic diversity of the area found that, a women's comprehension of her risk of developing breast cancer was associated with her ethnicity and this influenced screening attendance[7]. However a recent UK study using data from the women's health screening module of the National Statistics Omnibus Survey found no differences in attendance for breast screening between White British and the other ethnic groups combined, although White British women were significantly more likely to have had a cervical smear than were women in the other ethnic group[8].

Recent research in South East England has confirmed patterns of breast cancer incidence similar to those reported in the US, which show lower incidence in African American women than in White women in the screening age group[9]. Using self-assigned ethnicity from hospital episode statistics linked to cancer registry data, Jack and colleagues show that breast cancer incidence rates in non-White UK women are lower than for White women[10]. Adjusting for age and socioeconomic deprivation, Black Caribbean and Black African women are more likely to be diagnosed with advanced disease than White women. These women also have a lower age-adjusted breast cancer specific survival which is largely explained by advanced disease stage and socioeconomic deprivation[10].

The level of screening attendance among the different ethnic groups could be one of several factors affecting stage of breast cancer diagnosis and survival. Unfortunately, screening attendance cannot be directly calculated for different ethnic groups because although data on the ethnicity of women attending for screening is now being collected, data on the ethnicity of women receiving invitations are not yet available to the NHS. We have developed a method for assigning an individual's ethnicity based on their area of residence in order to estimate the association between cancer incidence and ethnicity (Ruth H. Jack, King's College London, personal communication, 2009). In this study, we used this method to investigate whether breast screening attendance differs between ethnic groups in London. Our objective was to produce estimates of the screening attendance for women in White, Black and Asian ethnic groups, taking into account other factors, including age and socioeconomic group.

## Method

### Data

We used records on 825,159 London women who had been invited to take part in the NHS Breast Screening Programme from April 2004 to March 2007. The data was collected by the London Cancer Screening Quality Assurance Reference Centre (QARC) from the six breast screening services in London. The variables included were date of birth, sex, postcode, screening service, type of appointment, and whether the women attended.

We excluded all interval cancer cases (6), non-routine appointments (490), GP referral appointments (4,776), self-referral appointments (39,593), other appointments (29) and males (66) from the analysis as these appointments are additional to the regular three yearly invitations provide by the NHS Breast Screening Programme. The invitations for appointments were further subdivided into two types: first call and the subsequent routine recall invitations.

The data set for analysis included 742,786 women between the ages 50 to 70 invited for screening at the six London breast screening services, North London (EBA), West London (ECX), Barking, Havering & Brentwood (FBH), Central & East London (FLO), South East London (GCA), and South West London (HWA). The primary care trusts (PCTs) covered by each of the London breast screening services are shown in Table 1. The services cover all of the 31 PCTs in the London Strategic Health Authority as well as six PCTs outside this area, namely Billericay, Brentwood, Wickford, Hertsmere, Watford and Three Rivers. These boundaries are current at the beginning of the study although minor changes in areas and names occurred during the three year period.

**Table 1:** Primary care trusts covered by the London breast screening services

Breast screening service	Primary care trusts
North London (EBA)	Enfield, Haringey, Barnet, Brent, Harrow, Hertsmere, Watford, Three Rivers

West London (ECX)	Ealing, Hammersmith & Fulham, Hounslow, Hillingdon, Kensington & Chelsea, Westminster

Barking, Havering & Brentwood (FBH)	Barking & Dagenham, Havering, Redbridge, Billericay, Brentwood, Wickford

Central & East London (FLO)	Camden, Islington, City & Hackney, Newham, Tower Hamlets, Waltham Forest

South East London (GCA)	Bexley, Bromley, Greenwich, Lambeth, Southwark, Lewisham

South West London (HWA)	Croydon, Kingston, Richmond & Twickenham, Sutton & Merton, Wandsworth

Cancer registries in England carry out cancer surveillance using the data they collect under Section 251 of the 2006 NHS Act. Their work includes routine exchange of data with the NHS Cancer Screening Programme which is subject to a protocol on confidentiality covering the collection, processing and release of data. The study used an anonymised dataset and separate ethical approval was not required.

### Assigning ethnicity

**Figure 1:**
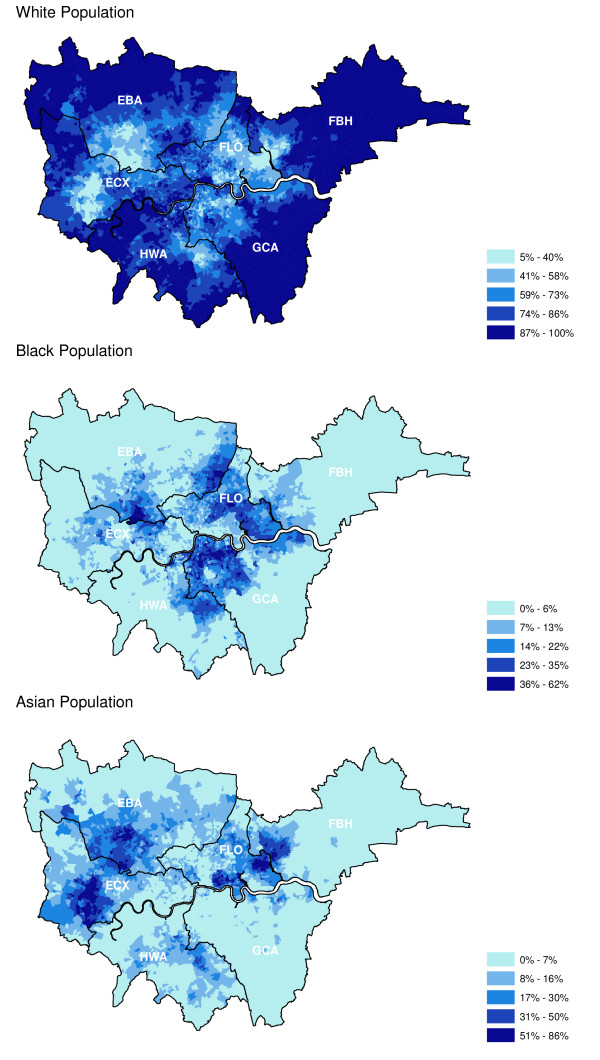
**Proportions of ethnic populations in the areas covered by the London breast screening units**.

We used an area-based method to estimate screening attendance by ethnicity. This method uses an individual's postcode of residence to assign them to a lower super output area (LSOA) which is a geographic area covering a population of approximately 1500 people. Information on the proportion of each ethnic group resident in each LSOA is available from the 2001 Census and was assigned to the women invited for screening. The ethnic groups in the 2001 Census are combined into three for these analyses so that the White group comprises White British, White Irish and other White, whilst the Black group comprises Black Caribbean, Black African and other Black. The Asian group consists of Indian, Pakistani, Bangladeshi and other Asian categories. The actual ranges of the proportions of the ethnic groups and their geographical distribution in six London breast screening services are shown in Figure 1.

### Analysis

We investigated screening attendance by age in the age groups 50-52, 53-54, 55-59, 60-64 and 65-70 years; by socioeconomic deprivation based on the income quintile of the Indices of Deprivation 2004[11], by invitation type and by breast screening service. Logistic regression was used to analyse screening attendance for each ethnic group. The ethnic group results were adjusted for age, socioeconomic deprivation, invitation type and screening service as these factors are known to cause variation in breast screening attendance. The estimated odds ratios express the attendance in a particular ethnic group compared with the other ethnic groups.

## Results

Table 2 shows the unadjusted attendance proportions and odds ratios for each age, socioeconomic deprivation, invitation type and screening service group. There was little difference in attendance between the age groups, but attendance varied considerably for the other variables. Attendance was highest for the most affluent socioeconomic group and fell sharply with increasing deprivation. Considering invitation type, attendance for routine recall was far higher than for the first call invitation. Variation in overall attendance ranged within the screening services from 54% for Central & East London (FLO) to 78% for Barking, Havering & Brentwood (FBH).

**Table 2:** Percentage attendance and odds ratio for attendance of breast screening in London, 2004-2007.

Age groups (years)	Number of women invited	% Attendance	Odds ratio	(95%CI)
50-52	60757	63.4	1.00	

53-54	91127	62.3	0.95	(0.93-0.98)

55-59	226705	61.7	0.93	(0.91-0.95)

60-64	202417	62.9	0.98	(0.96-1.00)

65-70	161780	61.0	0.90	(0.89-0.92)

**Socioeconomic deprivation**				

1 = Affluent	111016	69.2	1.00	

2	113994	67.5	0.92	(0.91-0.94)

3	140236	64.9	0.82	(0.81-0.84)

4	184006	61.1	0.70	(0.69-0.71)

5 = Deprived	192425	53.8	0.52	(0.51-0.53)

**Invitation type**				

First call	282998	40.1	1.00	

Routine recall	459788	75.6	4.64	(4.64-4.64)

**Screening service**				

North London (EBA)	146161	62.0	1.00	

West London (ECX)	148603	56.7	0.80	(0.79-0.81)

Barking, Havering & Brentwood (FBH)	61355	78.0	2.17	(2.12-2.22)

Central & East London (FLO)	98531	53.6	0.71	(0.70-0.72)

South East London GCA	151215	64.0	1.09	(1.07-1.10)

South West London (HWA)	136921	65.0	1.14	(1.12-1.15)

by age, socioeconomic deprivation, invitation type and screening service

Table 3 shows the odds ratios for breast screening attendance for each ethnic group. The unadjusted odds ratios were higher for the White group (OR: 3.34 95% confidence interval [3.26-3.42]) than for the Black group (OR: 0.13 [0.12-0.13]) and Asian group (OR: 0.55 [0.53-0.56]). Adjusting for age made no difference to the odds ratios for attendance in any of the ethnic groups but adjusting for socioeconomic deprivation and invitation type reduced the differences between them. Adjusting for the screening service attended increased the odds ratio for the Asian group to above unity. After adjustment for all variables, the odds ratio for the Black ethnic group remained below unity (OR 0.47 [0.44-0.50]).

**Table 3:** Odds ratios (OR) for breast screening attendance in White, Black and Asian ethnic groups, London 2004-2007.

Ethnic group	Unadjusted		Adjusted for age		and socioeconomic deprivation		and invitation type		and screening service	
	**OR**	**(95%CI)**	**OR**	**(95%CI)**	**OR**	**(95%CI)**	**OR**	**(95%CI)**	**OR**	**(95%CI)**

**White**	3.34	(3.26-3.42)	3.34	(3.26-3.42)	2.12	(2.06-2.18)	1.68	(1.62-1.73)	1.30	(1.26-1.35)

**Black**	0.13	(0.12-0.13)	0.13	(0.12-0.13)	0.35	(0.33-0.37)	0.50	(0.47-0.53)	0.47	(0.44-0.50)

**Asian**	0.55	(0.53-0.56)	0.55	(0.53-0.57)	0.73	(0.71-0.76)	0.82	(0.79-0.86)	1.10	(1.05-1.15)

unadjusted and adjusted for age, socioeconomic status and invitation type

There was considerable variation between the screening services in the odds ratios of attendance for the White, Black and Asian groups (Table 4). The unadjusted values for the Black group were below unity in all screening services. After adjusting for all variables, the Black group remained below unity in all screening services, except for Central & East London (FLO) (OR 1.65 [1.40-1.94]). For the Asian group, the unadjusted odds ratios for North London (EBA) and West London (ECX) were above unity and remained so after the adjustment for all variables. However, the opposite was true for screening services Barking, Havering & Brentwood (FBH), Central & East London (FLO) and South East London (GCA) where unadjusted odds ratios were below unity and remain so when adjusted for all variables. In South West London (HWA) the low unadjusted value for attendance of women in the Asian group rose to above unity after adjustment for all variables (OR 1.33 [1.09-1.62]). For the White group the unadjusted odds ratios for attendance of all the screening services were above unity and remained so after adjustment for the variables except for West London (ECX) (OR 0.94 [0.88-1.01]), although this difference does not reach statistical significance.

**Table 4:** Odds ratios (OR) for breast screening attendance for screening service in each ethnic group, London 2004-2007.

Screening service	Ethnic group	Unadjusted		Adjusted for age		and socioeconomic deprivation		and invitation type	
		**OR**	**(95%CI)**	**OR**	**(95%CI)**	**OR**	**(95%CI)**	**OR**	**(95%CI)**

**North London**	**White**	1.94	(1.84-2.05)	1.94	(1.84-2.05)	1.27	(1.20-1.36)	1.01	(0.94-1.09)

**(EBA)**	**Black**	0.14	(0.13-0.15)	0.14	(0.13-0.15)	0.22	(0.19-0.26)	0.34	(0.29-0.40)

	**Asian**	1.17	(1.08-1.26)	1.17	(1.08-1.26)	1.23	(1.14-1.33)	1.46	(1.34-1.60)

									

**West London**	**White**	1.11	(1.06-1.18)	1.12	(1.06-1.19)	1.00	(0.94-1.06)	0.94	(0.88-1.01)

**(ECX)**	**Black**	0.19	(0.16-0.23)	0.19	(0.16-0.22)	0.17	(0.14-0.22)	0.32	(0.25-0.42)

	**Asian**	1.48	(1.39-1.57)	1.47	(1.38-1.56)	1.54	(1.44-1.64)	1.44	(1.34-1.54)

									

**Barking, Havering**	**White**	3.24	(2.92-3.61)	3.18	(2.85-3.54)	2.50	(2.23-2.80)	2.89	(2.55-3.28)

**& Brentwood**	**Black**	0.004	(0.003-0.006)	0.005	(0.003-0.007)	0.009	(0.006-0.015)	0.010	(0.007-0.019)

**(FBH)**	**Asian**	0.28	(0.24-0.32)	0.29	(0.25-0.33)	0.37	(0.32-0.42)	0.29	(0.24-0.34)

									

**Central &**	**White**	2.62	(2.45-2.80)	2.61	(2.44-2.80)	1.93	(1.78-2.10)	1.50	(1.38-1.64)

**East London**	**Black**	0.61	(0.54-0.69)	0.60	(0.53-0.69)	1.59	(1.37-1.85)	1.65	(1.40-1.94)

**(FLO)**	**Asian**	0.42	(0.39-0.45)	0.42	(0.39-0.45)	0.54	(0.50-0.59)	0.67	(0.62-0.73)

									

**South East**	**White**	7.97	(7.50-8.46)	7.92	(7.45-8.41)	3.70	(3.39-4.03)	2.13	(1.94-2.35)

**London**	**Black**	0.09	(0.08-0.09)	0.09	(0.08-0.09)	0.23	(0.21-0.26)	0.45	(0.40-0.51)

**(GCA)**	**Asian**	0.07	(0.05-0.10)	0.07	(0.05-0.10)	0.30	(0.22-0.41)	0.52	(0.37-0.74)

									

**South West**	**White**	2.35	(2.18-2.54)	2.35	(2.18-2.54)	1.19	(1.09-1.31)	1.10	(1.00-1.22)

**London**	**Black**	0.20	(0.18-0.23)	0.20	(0.18-0.23)	0.74	(0.62-0.87)	0.78	(0.65-0.94)

**(HWA)**	**Asian**	0.58	(0.49-0.69)	0.58	(0.49-0.69)	1.17	(0.98-1.40)	1.33	(1.09-1.62)

unadjusted and adjusted for age, socioeconomic status and invitation type

## Discussion

### Summary of main findings

Using an area based method to assign ethnicity we found differences in estimates for attendance for breast screening between the White, Black and Asian groups in London between 2004 and 2007. There was little difference in attendance between age groups but attendance fell much more sharply with increasing socioeconomic deprivation. Attendance for routine recall appointments was also far higher than for the first call appointment. For the Black ethnic group the odds ratio of attendance was low and remained below unity after adjustment for all variables. In contrast, attendance for the Asian population was low but improved to above unity after adjustment for other variables. There were wide variations in attendance for different ethnic groups between the individual screening services.

### Limitations of the study

Historically, ethnicity has been poorly recorded in the UK and the individual ethnicity of the women in this study was unknown. We have used an area-based method where the ethnic composition for small geographical areas (LSOA) is known from the 2001 Census, and assigned this to each woman based on her postcode of residence. This is a similar method to that routinely used to derive area-based indicators of socioeconomic deprivation. A limitation of this method is that the extrapolation becomes unstable where the proportions of the ethnic groups are small, and for this reason we do not report the smaller groups of Mixed and Chinese ethnicities. Similarly, we combined categories for the White group, Black group and Asian group. The aggregation of these ethnic groups makes the estimates more robust but loses more specific information.

A further limitation is the reliance on ONS figures for the number of women in each ethnic group. These figures are an estimate based on the 2001 Census and may be conservative.

In addition, London has a highly mobile population making it difficult for general practices to maintain the accuracy of their lists[4], which are used to invite women for breast screening. A study exploring the effects of population mobility on cervical screening coverage in London[12] estimated that movement in and out of some boroughs could mean that up to 20% of the population changed each year. Women who do not update their details after moving will be sent invitations to their old address and so inflate non-attendance. The GP financing system tends to reinforce list inflation as income to the practise is lost when patients are deregistered. Our method assumes that the inaccuracies in the general practice administrative lists occur equally for each of the ethnic groups.

### Comparison to findings of previous studies

In our study there was little difference in screening attendance within the age groups, although attendance fell slightly as age increased, consistent with that reported in other studies[13-15]. Socioeconomic deprivation is known to have a very strong influence on screening behaviour. Our findings support this with women in the most deprived group being far less likely to attend for screening. The effect of deprivation on attendance for breast screening is difficult to separate from other factors including ethnicity, which influence attitudes to general health behaviour. Previous studies[14,16] have concluded that in addition to socioeconomic status other factors such as the neighbourhood non-attendance, being born abroad and aspects of health behaviour such as not visiting a dentist or doctor in the last 5 years, influence attendance for breast screening. As we expected, screening attendance was significantly higher in women who have previously attended as those that come for screening at first call are more likely to come back for subsequent routine invitations[17].

Few studies have investigated differences in attendance for screening in relation to ethnicity within a population-based breast screening programme. Our finding that the Black ethnic group is less likely to attend for screening is consistent with population studies conducted in the US[18,19], although there is also some evidence in the US that disparities between African American women and White women occurring in the 1990s, may have been reduced by efforts to improve access to screening services[20].

Studies of breast screening attendance in London have tended to be small, concentrating on particular localities and producing differing results. One published questionnaire-based study conducted on 306 women in South East London found that there were differences between ethnic groups in perceptions of breast screening[6]. Regular attendance was associated with ethnicity, although consistent avoidance of mammography was not. Black and minority ethnic groups were found to be ambivalent attenders for breast screening and were more likely to drop-out from the programme than White women[6]. By contrast, an earlier postal questionnaire survey, also in South East London found that Black women had a higher than average attendance although this relationship did not hold in a sample interviewed for the study[21]. A recent large UK study, using the National Statistics Omnibus Survey 2005-2007 found no significant differences in attendance between White British women and all the other ethnic groups combined[8]. Direct comparison with the results of this study is difficult because non-routine and routine screening were combined into an 'ever been screened' category for this analysis. Attendance was self-reported and will therefore be influenced by recall bias and limited by greater than 30% of the women selected not responding to the questionnaire[8]. Questionnaires and interviews are frequently used in studies concerning non-attendence for breast screening. A major problem with these methods is that the non-attenders are also likely to not respond to the questionnaire.

In our study the Asian group was initially less likely to attend for breast screening but after sequential adjustment for socioeconomic deprivation, invitation type and screening service this became less clear with this group becoming as likely to attend as the White group. Population-based studies in the Midlands[22] and West Yorkshire[23] using surnames to identify Asian women found that their breast screening attendance was lower than non-Asians. It should be remembered that the Asian group in our study includes Indian, Pakistani, Bangladeshi and Asian other categories defined in the 2001 Census. These ethnic groups have been developed for administrative purposes and give little information on faiths and cultures which may be a significant influence on behaviour. Differences within this Asian group are likely to produce very different screening behaviours. Our study suggests high attendance of breast screening in Asian women in the North London and West London screening services. These two areas have a higher proportion of Indian women than the other Asian groups in comparison to the other screening service areas. It is possible that the prominence of health professions including doctors from the Indian group in London has had an influence[24]. Furthermore, screening attendance for Asian women was seen to improve in the Midlands during 1989 to 2005 with the exception of the Muslim sub-group[22]. In contrast the attendance of Hindu-Gujarati women was similar to that of non- Asian women after adjusting for age and deprivation.

The influence of socioeconomic deprivation exerts a complex effect on attendance for screening. Our study shows wide variation in the attendance of the Asian group between the screening services and these disparities in attendance remain in three services even after adjusting for socioeconomic deprivation. This disparity disappears after the adjustment for socioeconomic deprivation in the South West London (HWA) screening service. Similarly, the disparity in Black women disappears after adjustment for deprivation in Central & East London (FLO) screening service. It is also possible that some of these differences reflect the ethnicity of healthcare professionals working within the screening service and the success of interventions to increase the participation of women from different groups.

### Implications for clinical practice, research and policy

The results of this study and several others from London[5,6] suggest that women belonging to Black ethnic groups are less likely to attend for breast screening. In addition, variations in attendance between the screening services are striking for the Asian group, after adjusting for the other variables. These differences require investigation at an individual screening service level to establish whether these differences are due to variations in the attendance of the Indian, Pakistani and Bangladeshi groups or possibly differences in practice in the screening services.

There is a need to encourage women from different backgrounds to attend screening. Strategies for increasing the participation could include many different forms of interventions using reminders by letter or telephone[25] as well as new social marketing techniques to improve awareness of breast cancer in the female population[26]. Provider interventions with feedback may also remind staff of targets. Both types of intervention have been shown to be effective and those that are culturally tailored tend to be more effective[27]. However there is little evidence on effective techniques for increasing the awareness of the benefits of breast screening and tailoring this message effectively to different ethnic and cultural groups. Research in this area might be promoted through the NHS, cancer charities and organisations focussing on the health of different ethnic groups.

## Conclusions

Overall breast screening attendance is low in communities with large Black populations, suggesting the need to improve participation of Black women. Variations in attendance for the Asian population require further investigation at an individual screening service level.

## Competing interests

The authors declare that they have no competing interests.

## Authors' contributions

CR contributed to the study's conception and design, analysed the data and wrote the first draft of the paper. RHJ contributed to the study design, helped analyse the data and write the paper. SD helped to design the study, interpret the findings and write the paper. HM helped interpret the findings and write the paper. ED contributed to the study's conception and design, interpret the findings and helped write the manuscript. All authors read and approved the final manuscript.

## Pre-publication history

The pre-publication history for this paper can be accessed here:

http://www.biomedcentral.com/1471-2458/10/157/prepub

## References

[B1] Forrest APM Breast cancer screening: Report to the Health Minister of England, Wales, Scotland and Northern Ireland 1986 London HMSO

[B2] Department of Health Cancer Reform Strategy 2007 Department of Health Publications, London

[B3] National Health Service Breast Screening Programme Breast Screening Programme England 2005-06 2007 National Statistics The Information Centre; Sheffield

[B4] Millett C Zelenyanszki C Binysh K Lancaster J Majeed A Population mobility: characteristics of people registering with general practices Public Health 2005 119 632 638 10.1016/j.puhe.2004.09.004 15885722

[B5] Greater London Authority Behind the screen: Breast screening uptake and radiotherapy waiting times in London 2008 Greater London Authority

[B6] Barter-Godfrey S Taket A Understanding women's breast screening behaviour: A study carried out in South East London, with women aged 50-64 years Health Educ J 2007 66 335 346 10.1177/0017896907083155

[B7] Pfeffer N Screening for breast cancer: candidacy and compliance Soc Sci Med 2004 58 151 160 10.1016/S0277-9536(03)00156-4 14572928

[B8] Moser K Patnick J Beral V Inequalities in reported use of breast and cervical screening in Great Britain: analysis of cross sectional survey data BMJ 2009 338 b2025 10.1136/bmj.b2025 19531549 PMC2697310

[B9] Smigal C Jemal A Ward E Cokkinides V Smith R Howe HL Trends in breast cancer by race and ethnicity: Update 2006 Ca-A Cancer Journal for Clinicians 2006 56 168 183 10.3322/canjclin.56.3.168 16737949

[B10] Jack RH Davies EA Møller H Breast cancer incidence, stage, treatment and survival in ethnic groups in South East England Br J Cancer 2009 100 545 550 10.1038/sj.bjc.6604852 19127253 PMC2658548

[B11] Neighbourhood Renewal Unit The English Indices of Deprivation 2004: Summary (revised) 2004 The office of the Deputy Prime Minister, London

[B12] Millett C Bardsley M Binysh K Exploring the effects of population mobility on cervical screening coverage Public Health 2002 116 353 360 10.1016/S0033-3506(02)00560-7 12407475

[B13] Zackrisson S Andersson I Manjer J Janzon L Non-attendance in breast cancer screening is associated with unfavourable socio-economic circumstances and advanced carcinoma Int J Cancer 2004 108 754 760 10.1002/ijc.11622 14696103

[B14] Lagerlund M Sparen P Thurfjell E Ekbom A Lambe M Predictors of non-attendance in a population-based mammography screening programme; socio-demographic factors and aspects of health behaviour Eur J Cancer Prev 2000 9 25 33 10.1097/00008469-200002000-00004 10777007

[B15] Banks E Beral V Cameron R Hogg A Langley N Barnes I Comparison of various characteristics of women who do and do not attend for breast cancer screening Breast Cancer Res 2002 4 R1 10.1186/bcr418 11879559 PMC83847

[B16] Zackrisson S Lindstrom M Moghaddassi M Andersson I Janzon L Social predictors of non-attendance in an urban mammographic screening programme: a multilevel analysis Scand J Public Health 2007 35 548 554 10.1080/14034940701291716 17852976

[B17] Tornberg S Kemetli L Svane G Rosen M Stenbeck M Nystrom L Pattern of participation in a cohort aged 50-60 years at first invitation to the service-screening programme with mammography in Stockholm county, Sweden Prev Med 2005 41 728 733 10.1016/j.ypmed.2005.07.004 16137757

[B18] Sassi F Luft HS Guadagnoli E Reducing racial/ethnic disparities in female breast cancer: Screening rates and stage at diagnosis Am J Public Health 2006 96 2165 2172 10.2105/AJPH.2005.071761 17077392 PMC1698161

[B19] Kagay CR Quale C Smith-Bindman R Screening mammography in the American elderly Am J Prev Med 2006 31 142 149 10.1016/j.amepre.2006.03.029 16829331

[B20] Sabatino SA Coates RJ Uhler RJ Breen N Tangka F Shaw KM Disparities in mammography use among US women aged 40-64 years, by race, ethnicity, income, and health insurance status, 1993 and 2005 Med Care 2008 46 692 700 10.1097/MLR.0b013e31817893b1 18580388

[B21] Sutton S Bickler G Sancho-Aldridge J Saidi G Prospective study of predictors of attendance for breast screening in inner London J Epidemiol Community Health 1994 48 65 73 10.1136/jech.48.1.65 8138773 PMC1059897

[B22] Szczepura A Price C Gumber A Breast and bowel cancer screening uptake patterns over 15 years for UK south Asian ethnic minority populations, corrected for differences in socio-demographic characteristics BMC Public Health 2008 8 346 10.1186/1471-2458-8-346 18831751 PMC2575216

[B23] Sutton GC Storer A Rowe K Cancer screening coverage of south Asian women in Wakefield J Med Screen 2001 8 183 186 10.1136/jms.8.4.183 11743034

[B24] Bedi R Gilthorpe MS Ethnic and gender variations in university applicants to United Kingdom medical and dental schools Br Dent J 2000 189 212 215 10.1038/sj.bdj.4800725a 11036749

[B25] Kearins O Walton J O'Sullivan E Lawrence G Invitation management initiative to improve uptake of breast cancer screening in an urban UK Primary Care Trust J Med Screen 2009 16 81 84 10.1258/jms.2009.009006 19564520

[B26] Evans WD How social marketing works in health care BMJ 2006 332 1207 1210 10.1136/bmj.332.7551.1207-a 16710002 PMC1463924

[B27] Valdez A Banerjee K Ackerson L Fernandez M A multimedia breast cancer education intervention for low-income Latinas J Community Health 2002 27 33 51 10.1023/A:1013880210074 11845940

